# Development and Initial Evaluation of the Caring Through COVID-19 Psychotherapy Group for Family Care Partners

**DOI:** 10.1093/geroni/igab046.2947

**Published:** 2021-12-17

**Authors:** Evan Plys, Kadija N Williams, Caitlin J Tyrrell, Rachel Weiskittle

**Affiliations:** 1 University of Colorado Denver, Denver, Colorado, United States; 2 VA Boston Healthcare System, Brockton, Massachusetts, United States; 3 VA Pacific Islands Healthcare System, Honolulu, Hawaii, United States; 4 VA Boston Healthcare System, Jamaica Plain, Massachusetts, United States

## Abstract

This project details the development and initial evaluation of a manualized psychotherapy support group for family care partners of persons living with dementia, specially designed to address pandemic-related stressors. The authorship team, consisting of clinical geropsychologists, developed a treatment manual based on existing protocols, such as: cognitive behavioral therapy for pandemic-related stress, grief management and ambiguous loss, and caregiver family therapy. The resulting 8-week Caring Through COVID-19 psychotherapy group was piloted in an outpatient mental health clinic via tele-mental health with six family care partners of persons living with dementia. All participants were women and spouses or partners of the care recipient; mean age was 70.5 (SD = 9.07). Preliminary data showed a non-significant and small reduction in depression (d = .22) and non-significant moderate reductions in caregiver burden (d = .52) and pandemic-related stress (d = .64). Moderate non-significant improvements were observed in general caregiver self-efficacy (d = .62) and self-efficacy for emotional regulation (d = .67). The majority of participants reported that the content of the group was novel (83%) and relevant (83%); the most utilized topics outside of the group were accepting emotions (100%) and challenging negative cognitions (83%). Overall, most participants were very or extremely satisfied with the group (67%). Additional data is currently being collected with another cycle of the group (n = 4). Preliminary findings suggest that the Caring Through COVID-19 group may be beneficial for supporting family care partners during the pandemic. Considerations for implementation and future plans for dissemination will be discussed.

